# Immunity to vector saliva is compromised by short sand fly seasons in endemic regions with temperate climates

**DOI:** 10.1038/s41598-020-64820-9

**Published:** 2020-05-14

**Authors:** Fabiano Oliveira, Ekaterina Giorgobiani, Anderson B. Guimarães-Costa, Maha Abdeladhim, James Oristian, Lamzira Tskhvaradze, Nikoloz Tsertsvadze, Mariam Zakalashvili, Jesus G. Valenzuela, Shaden Kamhawi

**Affiliations:** 1 Vector Molecular Biology Section, Laboratory of Malaria and Vector Research, National Institute of Allergy and Infectious Diseases, National Institutes of Health, Rockville, Maryland 20852 USA; 2 R. G. Lugar Center for Public Health Research, National Center for Disease Control and Public Health (NCDC), Kakheti Highway 99, 0198 Tbilisi, Georgia

**Keywords:** Genetic databases, Sequence annotation, Parasite host response

## Abstract

Individuals exposed to sand fly bites develop humoral and cellular immune responses to sand fly salivary proteins. Moreover, cellular immunity to saliva or distinct salivary proteins protects against leishmaniasis in various animal models. In Tbilisi, Georgia, an endemic area for visceral leishmaniasis (VL), sand flies are abundant for a short period of ≤3 months. Here, we demonstrate that humans and dogs residing in Tbilisi have little immunological memory to saliva of *P. kandelakii*, the principal vector of VL. Only 30% of humans and 50% of dogs displayed a weak antibody response to saliva after the end of the sand fly season. Likewise, their peripheral blood mononuclear cells mounted a negligible cellular immune response after stimulation with saliva. RNA seq analysis of wild-caught *P. kandelakii* salivary glands established the presence of a typical salivary repertoire that included proteins commonly found in other sand fly species such as the yellow, SP15 and apyrase protein families. This indicates that the absence of immunity to *P. kandelakii* saliva in humans and dogs from Tbilisi is probably caused by insufficient exposure to sand fly bites. This absence of immunity to vector saliva will influence the dynamics of VL transmission in Tbilisi and other endemic areas with brief sand fly seasons.

## Introduction

Visceral leishmaniasis (VL) is a neglected disease transmitted by *Leishmania*-infected sand fly bites (SFB) and is fatal if left untreated^1^. The burden of VL is estimated at 500,000 new cases annually, and the disease is only third after malaria and cryptosporidium for parasitic-induced mortality^2^. The contiguous districts of Mtatsminda and Vake in Tbilisi, Georgia are considered as an active VL focus, with high infection rates in canines and humans^3^. *Phlebotomus kandelakii* has been incriminated as the main vector of VL in this region^4^. Seasonality of sand flies, including *P. kandelakii*, in Tbilisi, Georgia, is of short duration, and sand flies are only abundant for ≤3 months of the year^4,5^.

It has been established that previous exposure to uninfected SFB protects mice and non-human primates from a subsequent challenge by infected SFB^6,7^. Protection is attributed to a saliva-specific cellular immune response that adversely impacts *Leishmania* development after co-deposition of saliva and parasites at the bite site during a blood meal^8^. The salivary proteins, PpSP15 and its homologue PdSP15, from Old World sand flies *Phlebotomus papatasi* and *P. duboscqi*, respectively, and LJM19 and LJM11 from the New World sand fly *Lutzomyia longipalpis*, vectors of cutaneous leishmaniasis (CL) and VL, respectively, induce a T_H_1-biased cellular immunity, are protective against leishmaniasis, and are considered as promising vaccine candidates among others as, reviewed by Abdeladhim *et al.*^8^. PpSP15 and LJM11 conferred protection against *Leishmania major* in mice. Similarly, LJM19 protected hamsters against *Leishmania infantum*^9^ and *Leishmania braziliensis*^10^. Additionally, PdSP15 protected non-human primates against sand fly-delivered *L. major*^11^. Others such as PpSP32 from *P. papatasi*^12^, mYEL1 and mAG5 from *P. orientalis*^13^, and LJM17 and LJM11 from *L. longipalpis*^14^, induce a strong antibody response and have been used as biomarkers of sand fly exposure in humans^15^. Pertinently, a robust immune response has been documented in humans naturally exposed to sand fly bites in Mali, Western Africa, where humans are exposed to uninfected SFB throughout the year^16–19^.

Thirteen transcriptomic studies of salivary protein repertoires from several sand fly vectors of leishmaniasis revealed at least 12 salivary protein families shared between New World and Old World sand flies^20^. Salivary proteins common to most sand fly species include the yellow, SP15, Lufaxin, Antigen 5, Silk and Apyrase families among others^20^. These include molecules with various known pharmacological functions such as anti-inflammatory, anti-complement, and pro-vasodilation^8^. To date, the function of several families has not been ascribed^8^.

Here, we demonstrate that a short sand fly season does not induce a lasting immune response to *P. kandelakii* salivary proteins in humans and dogs residing in Tbilisi. We also characterize the protein repertoire from salivary glands of wild-caught *P. kandelakii* using our in-house custom de novo transcriptome analysis of the RNA-seq dataset generated using a HiSeq illumina platform and validate its composition. This work provides an insight into the significance of salivary proteins of vector sand flies in areas of short sand fly seasonality. Moreover, it provides a catalogue of the salivary proteins of *P. kandelakii*, and a path towards identifying molecules that have potential for future use as biomarkers for *P. kandelakii* exposure or as potential vaccine candidates for VL in areas where this vector species is present.

## Results and Discussion

### The immune response to *P. kandelakii* saliva in humans and dogs residing in Tbilisi, Georgia

**Figure 1:**
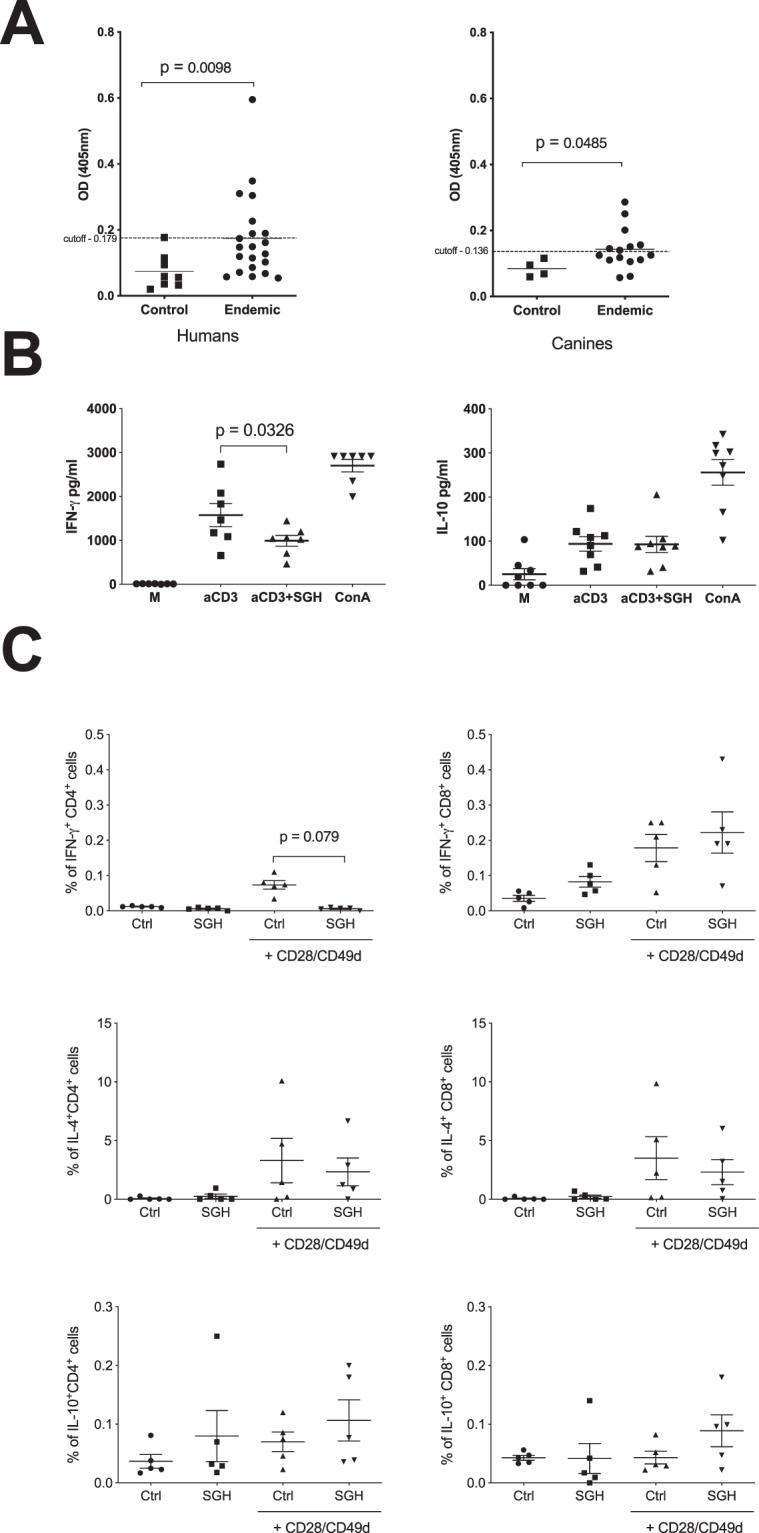
The immune response to *P. kandelakii* saliva in humans and dogs from Tbilisi. (**A**) IgG antibodies to *P. kandelakii* salivary gland homogenate (SGH) were investigated in 21 humans and 14 dogs living in an endemic area of visceral leishmaniasis in Tbilisi compared to 8 US volunteers and 4 US dogs naive to sand fly bites. The cut-off was determined as mean OD of controls plus 2 SD, n = 29 (**B**) Supernatants of human PBMC cultures unstimulated or stimulated with anti-CD3 alone, anti-CD3 together with *P. kandelakii* saliva (SGH), or Concavalin A (ConA) as a positive control, n = 7. (**C**) Human PBMC were stimulated with *P. kandelakii* SGH in the presence or absence of CD28/CD49d. The frequency of CD4 cells producing IFN-γ, IL-10 and IL-4 was measured by flow cytometry, n = 5. Lines in scatterplots represent the mean and error bars the standard error of the mean.

Human and dog sera were collected during August 2013, towards the end of the sand fly season. The collected sera were examined for specific IgG antibodies against *P. kandelakii* salivary proteins by ELISA. Compared to controls, we detected a statistically significant increase in antibodies to salivary proteins in sera of humans, and in dogs (Fig. 1A). Surprisingly, only 30% of humans and 50% of dogs had anti-*P. kandelakii* salivary antibodies above the calculated cut-off. This frequency is low compared to other vector saliva antibody surveys in leishmaniasis endemic areas^21–24^. In a cohort from India and Nepal, antibodies against *P. argentipes* saliva were present in 63.2% subjects^21^. Moreover, an 83% positivity against *P. papatasi* saliva and a 53% positivity against *L. intermedia* saliva were reported for humans living in Tunisia and Brazil, respectively^22,23^. Similarly, 55% to 88% of canines in a leishmaniasis endemic area in Italy were positive against *P. pernicious* saliva^24^.

Though antibodies against vector saliva are useful markers of vector exposure^25^, sand fly saliva-specific protection from leishmaniasis in animal models is cell-mediated^26^. Therefore, we investigated the response to *P. kandelakii* salivary gland homogenate (SGH) in peripheral blood mononuclear cells (PBMC) obtained from human volunteers and dogs residing in Tbilisi. PBMC stimulated with *P. kandelakii* SGH were tested for human IFN-γ, IL-10, IL-17, IL-13, IL-5, IL-9, IL-2 and IL-4, or for canine IFN-γ, IL-10, IL12p40, TNF-α and IL-6 cytokines by Luminex. Surprisingly, stimulation with *P. kandelakii* SGH did not induce any of the tested cytokines (Supplemental Fig. 1).

In naïve individuals lacking an adaptive immune response to sand fly saliva, immunomodulatory salivary proteins have been shown to be mostly anti-inflammatory^8^. To investigate whether *P. kandelakii* saliva has immunosuppressive properties, we stimulated PBMC from our study subjects with anti-CD3 in the presence or absence of *P. kandelakii* SGH. This resulted in a significant decrease in IFN-γ levels and no change in IL-10 levels compared to PBMC stimulated with anti-CD3 alone (Fig. 1B). Further, flow cytometric analysis of PBMCs stimulated with SGH in the presence of CD28/CD49d co-stimulatory molecules resulted in a statistically significant reduction in the frequency of IFN-γ-producing-CD4^+^ T lymphocytes compared to PBMC stimulated with CD28/CD49d alone (Fig. 1C). The addition of SGH did not alter the frequency of CD8^+^ T cells producing IFN-γ, nor the frequency of either CD4^+^ or CD8^+^ T cells producing IL-10, TNF-α and IL-4 cytokines compared to controls (Fig. 1C). These results contrast with data from individuals living in other sand fly prevalent areas where the sand fly season is longer. In Mali, stimulation with *P. duboscqi* SGH induced measurable levels of IFN-γ, IL-12, IL-10, IL-13 and IL-5^27^; in Tunisia stimulation with *P. papatasi* SGH induced IL-4 and IL-10 production^28^; and in Brazil stimulation with *L. intermedia* SGH induced mostly IL-10 production followed by IL-13 and IFN-γ^23^. The lack of IL-10 production after stimulation of PBMC with *P. kandelakii* saliva also emphasizes its distinct immunomodulatory effect compared to other vectors such as *P. papatasi* and *P. argentipes* where the presence of adenosine promotes blood feeding and induces IL-10 production in healthy human monocytes^29–31^. Overall, the weak humoral and absent adaptive cellular immune response in both humans and dogs suggests that the brief sand fly season in Tbilisi^4,5^ is not sufficient to generate a robust memory response to *P. kandelakii* saliva. Furthermore, the relative low density of sand flies during the sand fly season^4,5^, suggests that a low biting rate may also have contributed to the observed weak immune response to *P. kandelakii* saliva. Therefore, we hypothesize that upon an encounter with an infected *P. kandelakii* sand fly, Tbilisi individuals would not benefit from immunity to sand fly saliva and would therefore be more prone to infection. In this scenario, exposure to *P. kandelakii* saliva during blood feeding would enhance their susceptibility to infection through its immunosuppressive effect, demonstrated by a reduction in the percent of CD4^+^-IFN-γ-producing cells, though its effect on other cell types also needs to be elucidated. It is important to note that under these conditions, finding molecules in *P. kandelakii* saliva that induce a Th1 response in humans or dogs through vaccination remains a valid and effective strategy to mitigate vector-transmitted leishmaniasis. Of note, these findings may have broader implications for leishmaniasis foci in other countries where the sand fly season is short. Comparative analyses in areas with similar climate and sand fly seasonality are needed to further support our conclusions.

### Insights into the repertoire of *P. kandelakii* salivary proteins

To date there is no information regarding the salivary gland transcriptome of *P. kandelakii*. Therefore, we applied a de novo assembled transcriptome analysis of RNA-seq from salivary glands of wild-caught sand flies to identify the repertoire of *P. kandelakii* secreted salivary proteins. Besides the potential benefit of the generated data for control strategies, it also provides an opportunity to compare the *P. kandelakii* salivary repertoire with those available for other vector species. Moreover, it serves to ascertain that the observed absence of immunological responses in humans and canines (Fig. 1) is not caused by deficiencies in the makeup of *P. kandelakii* saliva.

De novo transcriptome assembly of 6.27 ×10^7^ high quality HiSeq illumina reads resulted in 6049 coding sequences (CDS) that were first organized in broad categories as putatively secreted, housekeeping, transposable elements, viral products and unknown sequences (Table 1). Calculation of the reads per kilobase of transcript per million mapped reads (RPKMs) allowed us to quantify sand fly saliva CDS for the first time, since previous data from reported sand fly salivary transcriptomes were generated by conventional non-normalized cDNA libraries and low-output sequencing^32–41^. Corroborating the secretory nature of salivary glands, the majority of CDS (87.28%) contained a signal peptide motif and were classified as putative secreted proteins. They spread out to 501 unique contigs generating 2322 RPKM/contig. Housekeeping CDS mapped to 9.68% of the RPKM represented by 4322 contigs (30 RPKM/Contig) followed by “unknown” representing 2.68% of the reads. Transposable elements and viral CDS were marginally detected at 0.03% and 0.02% of RPKM abundance, respectively.

**Table 1:** Classification of the putative coding sequences (CDS) from P. kandelakii salivary glands.

Categories	# Contigs	RPKM	% Contigs	% RPKM
Putative Secreted	501	1163684	8.28%	87.28%
Housekeeping	4322	130987	71.45%	9.82%
Unknown	1148	37906	18.98%	2.84%
Transposable element	50	388	0.83%	0.03%
Viral product	28	266	0.46%	0.02%
Total	6049	1333231	100	100

The previously reported, low output, sand fly salivary gland transcriptomes resulted in identification of putative secreted salivary proteins from thirteen different sand fly vectors from several geographical regions, reviewed by Abdeladhim *et al.*^8^. Taking advantage of the quantitative data acquired in this work, we set an expression threshold of two-folds the average RPKMs in the global transcriptome (441 RPKM) to rank secreted salivary proteins by abundance. Using this parameter, we ranked 18 of 43 secreted salivary protein families as the most abundantly secreted *P. kandelakii* salivary proteins (Table 2 – high abundance). Strikingly, three proteins represented 57.3% of all generated RPKM and 65.4% of all the putative secreted CDS in *P. kandelakii* saliva. The most represented CDS were the yellow family of proteins at 31.7% of secreted transcripts (368,804 RPKM) followed by the SP15-family of sand fly salivary proteins. The SP15-family of proteins comprised 20.88% of all secreted proteins (243,022 RPKM). The Apyrase protein family was the third most abundant, represented by 13.07% of CDS (152,125 RPKM). These sand fly salivary protein families have been found in all sand fly salivary transcriptomes available to date^8^. Future studies comparing the makeup of the sand fly salivary glands by RNAseq in different species will help establish possible correlations with vector competence and disease patterns.

**Table 2:** Classification of the putative secreted salivary proteins of P. kandelakii.

Secreted proteins	Contigs	RPKM	RPKM/ Contig	% of RPKM
*High Abundance*				
PkanYLW -family	8	368804	46100.5	31.6928
PkanSP15-family	5	243022	48604.4	20.8838
Apyrase	6	152125	25354.2	13.0727
D7-like	3	85470	28490	7.3448
37 kDa-family	2	81577	40788.5	7.0102
Antigen 5-family	5	50983	10196.6	4.3812
PkanSILK	1	47049	47049	4.0431
Lufaxin-like	1	46129	46129	3.964
5 kDa-family	1	22946	22946	1.9718
ParSP25-like	1	19711	19711	1.6938
Hyaluronidase	2	9212	4606	0.7916
Amylase	2	4916	2458	0.4225
Pyrophospatase	2	4421	2210.5	0.3799
Phospholipase A2	1	3221	3221	0.2768
Endonuclease	10	3214	321.4	0.2762
PArSP13-like	1	2841	2841	0.2441
PAbSP107-like	1	2022	2022	0.1738
*Low Abundance*				
Serine Protease inhibitors	12	842	70.2	0.0724
SP14-like	6	496	82.7	0.0426
Cathepsins	28	475	17	0.0408
SP5-like	1	308	308	0.0265
Apolipoprotein	2	245	122.5	0.0211
16 kDa family	2	226	113	0.0194
32.2 kDa Lipase	6	108	18	0.0093
SP10	2	68	34	0.0058
Mucin-like	10	67	6.7	0.0058
OBPs	3	66	22	0.0057
Metaloprotease inhibtor	1	64	64	0.0055
17 kDa -family	1	60	60	0.0052
SP29 (Neuroplastin)	1	45	45	0.0039
Mucins	2	44	22	0.0038
SP12-like	1	42	42	0.0036
SP24-like	1	39	39	0.0034
Carboxypeptidase	2	38	19	0.0033
Vasotab	2	34	17	0.0029
Lectins	2	29	14.5	0.0025
SP13-like	1	26	26	0.0022
SP6-like	1	18	18	0.0015
Metalloprotease	2	18	9	0.0015
Kazal-type	1	17	17	0.0015
Lipocalin	3	13	4.3	0.0011
SP71-like	1	4	4	0.0003
Unknown Secreted	355	12629	35.6	1.0853

The second tier of abundant CDS were composed of D7 proteins (7.35%), the 37 kDa family (7.01%), the Antigen-5 family (4.38%), the Collagen binding-like (PkanSILK) family (4.04%), Lufaxin-like proteins (3.96%), the 5 kDa family (1.97%), ParSP25-like protein (1.69%), Hyaluronidase (0.79%), Amylase (0.42%), Pyrophosphatase (0.38%), Phospholipase A2 (0.28%), Lundep-like (0.28%), PArSP13-like (0.24%) and PAbSP107-like (0.17%). Twenty-five additional families were identified (Table 2- low abundance), but with very low representation in the transcriptome (<0.1% RPKM abundance). Due to the massive amount of data generated, we ran targeted blast analysis looking for transcripts found in saliva of other sand fly species but are absent from the *P. kandelakii* dataset. Sand fly salivary families previously identified only in saliva of New World sand flies from the genus *Lutzomyia* such as C-type lectins and RGD-containing family of proteins were absent from the *P. kandelakii* transcriptome^34,36,42^. Similarly, Maxadilan, a potent vasodilator and a vaccine candidate against leishmaniasis that is abundantly in *L. longipalpis* salivary glands^43^, was not detected here. Adenosine deaminase (ADA) an enzyme that hydrolyses adenosine to inosine^44^ reported to date from the salivary transcriptomes of *P. duboscqi* and *L. longipalpis* was also absent from the *P. kandelakii* analysis. The absence of ADA may be indicative of the presence of adenosine in *P. kandelakii* saliva, as reported for *P. papatasi* and *P. argentipes*^45,46^. The presence of adenosine remains to be confirmed by biochemical analysis of *P. kandelakii* saliva. Interestingly, the 5kDa-family present in *P. kandelakii* (Table 2 - 1.9% of the RPKM) has so far been only identified in sister sand fly species of the subgenus *Larroussius*, namely *P. tobbi, P. pernicious and P. ariasi*^37,39,40^. Taken together, the *P. kandelakii* repertoire of salivary proteins is similar in diversity to those previously reported for other sand fly species^8^. This indicates that the absence of immunity to saliva of *P. kandelakii* in Tbilisi is not caused by a deficiency in the composition of its saliva but is likely the outcome of the short period in which humans and dogs are exposed to bites of this vector each year.

Since a proportion of both humans and canines living in Tbilisi developed antibodies to *P. kandelakii* saliva (Fig. 1A), identifying biomarkers of vector exposure can be useful to determine their exposure to vector bites before, during and after a sand fly season as a marker of risk for developing VL. Using recombinant salivary proteins instead of *P. kandelakii* SGH as biomarkers of sand fly exposure can be advantageous. Using a defined antigen overcomes technical difficulties, such as capturing sufficient sand flies in such a short season and insures better reproducibility. To date, five sand fly recombinant salivary proteins from various species have been validated as markers of human exposure to sand fly bites in leishmaniasis endemic areas. rPpsp32, a salivary protein from *P. papatasi*, was tested in Tunisia and Saudi Arabia where *P. papatasi* is the main vector of CL^12,22,47,48^, Antigen 5 and a yellow protein from *P. orientalis* were tested on human sera from Sudan and Ethiopia^13^, and yellow proteins, rLJM11 and rLJM17 from *L. Longipalpis* saliva, the main vector of VL in South America were tested in Brazil^49,50^. Additionally, PpSP32 and the combination of LJM11/LJM17 were validated in large cohorts in Tunisia and Brazil, respectively, as feasible SGH substitutes^12,49^.

**Figure 2:**
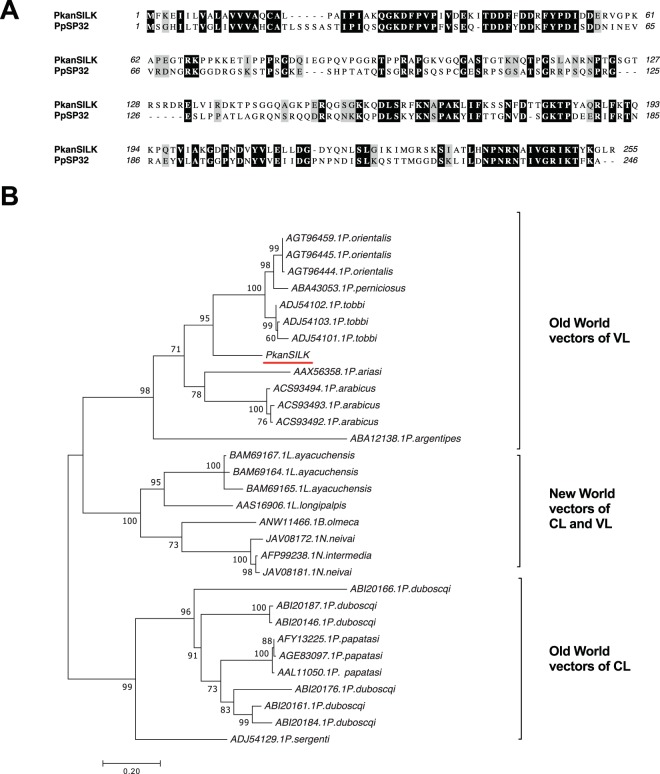
Sequence alignment and molecular phylogenetic analysis of the PkanSILK protein. (**A**) Protein sequence alignment of PpSP32 and PkanSILK using Muscle. Black shading and gray shading represent identical and similar amino acids, respectively. (**B**) The evolutionary history was inferred by using the Maximum Likelihood method based on the JTT matrix-based model^56^. The tree with the highest log likelihood (−4268.21) is shown. The percentage of trees in which the associated taxa clustered together is shown above the branches. The tree is drawn to scale, with branch lengths measured in number of substitutions per site. The analysis involved 31 amino acid sequences. All positions with less than 95% site coverage were eliminated. Evolutionary analyses were conducted in MEGA7^57^.

In the salivary transcriptome of *P. kandelakii*, we found a homologue belonging to the silk family of proteins, PkanSILK (GIFK01005859). PkanSILK shares 30% identity and 46% similarity to PpSP32, another member of the silk family of proteins (Fig. 2A). Due to the low identity, PpSP32 may be a weak marker of exposure to *P. kandelakii*, but it provides a rationale to prioritize the production and testing of a recombinant PkanSILK for that purpose. Phylogenetic analysis of the silk protein family from several species shows segregation of members by geographical region (Old World and New World vectors) and by their ability to cause VL or CL in the Old World (Fig. 2B). Based on this distribution and on sequence identity, we hypothesize that PkanSILK may be a good marker of exposure to other vectors including *P. orientalis* (66% identical)*, P. tobbi* (66% identical) and *P. perniciosus* (58% identical). Interestingly, PpSp32 antibody levels are increased in CL subjects compared to non-diseased endemic subjects, suggesting it may also be a marker of risk for acquiring disease^48^. Of note, the function of the silk family of proteins remains unknown.

**Figure 3:**
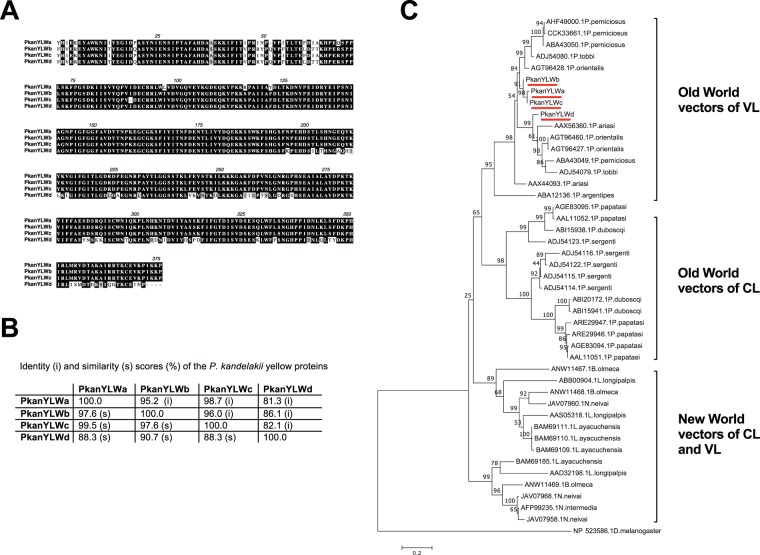
Sequence alignment and molecular phylogenetic analysis of the PkanYLW proteins. (**A,B**) Protein sequence alignment and similarity and identity percentages of PkanYLWs using Muscle. Black shading and gray shading represent identical and similar amino acids, respectively. (**C**) The evolutionary history was inferred by using the Maximum Likelihood method based on the Whelan and Goldman model^58^ The tree with the highest log likelihood (−11041.70) is shown. The percentage of trees in which the associated taxa clustered together is shown above the branches. The tree is drawn to scale, with branch lengths measured in number of substitutions per site. The analysis involved 45 amino acid sequences. All positions with less than 95% site coverage were eliminated. Evolutionary analyses were conducted in MEGA7^57^.

*P. kandelakii* yellow proteins were the most identified CDS in the transcriptome (31.7% of the RPKM). We found four abundant homologues namely PkanYLWa (GIFK01005845), PkanYLWb (GIFK01005846), PkanYLWc (GIFK01005847), PkanYLWd (GIFK01005848) that are at least 81.3% identical by sequence alignment (Fig. 3A,B). All the yellow proteins show at least 40% identity, being remarkably conserved across species (Supplemental Table 1). Phylogenetic analysis of the yellow family also clusters sequences by geographical location for Old and New World sand flies and by vectorial competence for VL or CL (Fig. 3C). The yellow proteins in the sand fly vector *L. longipalpis* have been identified as molecules able to bind biogenic amines such as histamine and serotonin, altering hemostasis and inflammation at the bite site^51^. The immunogenicity of PkanSILK and PkanYLWs in Tbilisi inhabitants needs to be assessed to determine the better candidate for monitoring vector exposure in the population.

**Figure 4:**
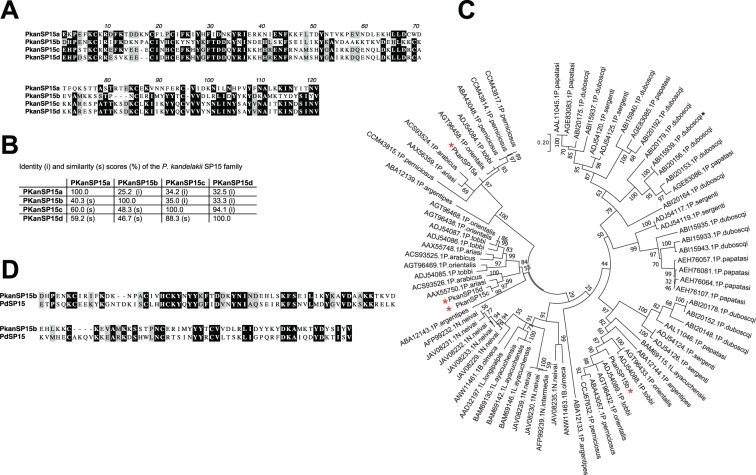
Sequence alignment and molecular phylogenetic analysis of the PkanSP15 proteins. (**A,B**) Protein sequence alignment and similarity and identity percentages of PkanSP15 using Muscle. Black shading and gray shading represent identical and similar amino acids, respectively. (**C**) The evolutionary history was inferred by using the Maximum Likelihood method based on the Whelan and Goldman model^58^. The tree with the highest log likelihood (−8196.26) is shown. The percentage of trees in which the associated taxa clustered together is shown above the branches. The tree is drawn to scale, with branch lengths measured in protein sequences number of substitutions per site. The analysis involved 78 amino acid sequences. All positions with less than 95% site coverage were eliminated. Evolutionary analyses were conducted in MEGA7^57^. Red asterisks highlight PkanSP15 proteins and the black asterisk the PdSP15 protein. (D) Protein sequence alignment of PdSP15 and PkanSP15 using Muscle. Black shading and gray shading represent identical and similar amino acids, respectively.

In addition to finding a good marker of vector exposure, having the salivary transcriptome of *P. kandelakii* may also help identify potential vaccine candidates against VL transmitted by this sand fly species. Repeated uninfected SFB, or immunization with either SGH or recombinant salivary proteins that produce a Th1 immune response have been shown to protect rodents against a subsequent *Leishmania* infection^52^. Additionally, immunization of rhesus macaques with the salivary protein PdSP15 from *P. duboscqi* protected monkeys against a virulent *L. major*-infected *P. duboscqi* challenge^7^. Further, PBMC from individuals living in an endemic area of CL where *P. duboscqi* is prevalent responded to *in vitro* stimulation with recombinant PdSP15 producing IFN-γ and IL-17 at levels comparable to those produced in response to SGH^7^. Though the role of IL-17 remains controversial^53^, IFN-γ is strongly associated to protection against leishmaniasis, reviewed by Kima^54^. In the *P. kandelakii* salivary transcriptome, four abundant putative proteins (PkanSP15a (GIFK01005849), PkanSP15b (GIFK01005850), PkanSP15c (GIFK01005851) and PkanS15d (GIFK01005852)) belong to the SP15 family. The four SP15 homologues in *P. kandelakii* saliva share poor identity (25–35%) (Fig. 4A,B) but each is notably more conserved among corresponding homologues in different sand fly vector species as shown by their distribution within the phylogenetic tree **(**Fig. 4C***)***. Possibly, three distinct SP15 sub-families of proteins were derived from a common distant ancestor, but with multiple events of gene duplication in different species, the disparities among sub-families were exacerbated. Interestingly, the structure of an ancestral odorant binding protein has been maintained by the presence of conserved cysteines hinting that the three different SP15 sub-families may all have a similar function. PdSP15 from *P. duboscqi* binds anionic surfaces such as heparin and polyphosphates, possibly preventing anti-hemostatic and anti-inflammatory processes^55^. Of the four PkanSP15 family of proteins, PkanSP15b shares the highest identity (34.4%) and similarity (51.6%) to PdSP15 (ABI15933.1) ***(***Fig. 4D***)***. At this low level of identity, we cannot speculate whether PdSP15 would cross-protect against a *P. kandelakii-*transmitted infection. Nevertheless, assessing the immunogenicity of PkanSP15b in humans, as well as screening for other Th1-inducing molecules in *P. kandelakii* saliva, should be undertaken.

In summary, this study reveals that humans and dogs residing in Tbilisi, Georgia, develop a weak immune response to *P. kandelakii* saliva, explained by a short sand fly season. We do not have an objective measure of the *P. kandelakii* biting frequency in Tbilisi, and a low biting rate could also be involved in the weak immune response to *P. kandelakii* saliva. The absence of a lasting immune response in Tbilisi inhabitants emphasizes the value of mining markers of exposure and vaccine candidates from vector saliva for use as powerful tools for leishmaniasis control in this focus. This is facilitated the availability of the transcriptome we have assembled of the salivary protein repertoire of *P. kandelakii*, the major vector of VL in this area. This is the first report to highlight the significance of the duration of the sand fly season in the development of saliva-specific immunity and should be taken into consideration in colder endemic regions with brief sand fly seasons.

## Materials and methods

### Study population

This study was conducted in the districts of Mtatsminda and Vake, Tbilisi, Georgia, considered as an active focus of VL^3^. Blood was collected from 21 human volunteers and 14 dogs were investigated. All human and animal study protocols were approved by the Ethics Committee of National Center for Disease Control and Public Health and were conducted according to and in compliance with the Georgian legislation and international norms in clinical and biological research. All human subjects provided written informed consent authorizing the cryopreservation and analysis of the blood derived samples.

### Sand fly capture

*Phlebotomus kandelakii* sand flies were collected throughout one sand fly season. Ten to 14 light traps were placed in productive sites identified from a previous study (Giorgobiani *et al.* 2012). A minimum of 10 light traps were used for two consecutive nights on a weekly basis from May to August 2013. Captured sand flies were provided with pieces of cotton soaked in a 30% sugar solution over a 3-day period to insure maturity of their salivary repertoire. Thereafter, *P. kandelakii* females were identified through characteristics of the spermatheca and pharynx using the dichotomous keys of Artemiev and Neronov (1984) and Artemiev (1974) prior to dissection of the salivary glands^59,60^.

### Salivary gland homogenate preparation

Female *P. kandelakii* salivary glands were dissected, placed in 20 µl of PBS and stored at −70 °C until needed. Salivary Gland Homogenate (SGH) was prepared by ultra-sonication followed by centrifugation at 10,000 g for 3 minutes at 4 °C. Supernatants were collected and stored at −80 °C until use.

### Blood collection and storage

Blood samples from human and dogs were collected in heparinized Vacutainer tubes (BD Diagnostics, Hunt Valley, MD). Human blood was collected from October 30^th^, 2013 to January 09^th^, 2014, while canine blood samples were all collected in the month of October 2013.

Peripheral blood mononuclear cells (PBMCs) were isolated by density-gradient centrifugation using a Ficoll-Paque PLUS solution (GE Healthcare, Pittsburgh, PA). Plasma was separated from the top layer and stored at −80 °C. Cells were then counted and frozen in a fetal bovine serum with 10% dimethylsulfoxyde solution (FBS-DMSO 10%) at −80 °C overnight, before they were transferred to liquid nitrogen.

### Detection of antibodies against saliva

Specific IgG antibodies to *P. kandelakii* saliva were assessed by ELISA. Ninety-six-well high binding microtiter plates (Thermo scientific, Rochester, NY, USA) were coated with 50 µl of SGH diluted to (1 pair/ml) in 0.1 M carbonate-bicarbonate buffer overnight at 4 °C. The wells were then washed in Tris-buffered saline (TBS) added with 0.05%Tween 20 and incubated with TBS–4% bovine serum albumin (BSA) for 1 hour at room temperature (RT) to block free binding sites. After three washes 50 µl of diluted Plasma (1:100) were added and incubated for 1 hour at 37 °C. Antibody-antigen complexes were detected using alkaline phosphatase-conjugated goat anti-human IgG (H + L) antibodies (Sigma, St Louis, MO) diluted at 1:5000 for 1 hour at RT and were visualized using nitrophenyl phosphate liquid substrate system (Sigma). The absorbance was measured at 405 nm using a Versamax microplate reader (Molecular devices, San Jose, CA). The cut-off for the assays was the mean optical density obtained with sera of eight negative controls plus three standard deviations.

### Cell culture and reagents

In all *in vitro* assays, cells were cultured in RPMI 1640 medium supplemented with 10% AB human serum (Sigma), 1% sodium pyruvate, 1% nonessential amino acids, 1% HEPES buffer, 5×10^−5^ M beta-mercaptoethanol and 40 mg/ml Penicillin/streptomycin. Frozen cells were taken out of liquid nitrogen, rapidly thawed at 37 °C before they were washed in RPMI solution for 10 min at 1400 rpm. Cells viability was assessed using Trypan blue. Cells were then cultured in 96-well plates in cell culture medium at 1×10^6^ cells/ml in a final volume of 200 µl and incubated with SGH (0.5 pair/ml), anti-CD3 (CD3 clone HIT3a, 555336), SGH + anti-CD3 or Concanavalin A (2.5 µg/ml) in a 5% CO2 humidified atmosphere at 37 °C as previously described^28^.

### Cytokine detection

For IFN-γ and IL-10 detection, supernatants of cell culture were collected after 96 h, centrifuged and stored at −80 °C until use. Capture enzyme-linked immunosorbent assay (ELISA) was performed on supernatants using anti-Human IFN-γ or anti-Human IL-10 ELISA Sets (BD Biosciences) according to manufacturer’s instructions. The results were interpolated from a standard curve using recombinant cytokines and expressed as ratio of cytokine concentration in stimulated/unstimulated cultures. Levels of human IFN-γ, IL-10, IL-17, IL-13, IL-5, IL-9, IL-2 and IL-4, or canine IFN-γ, IL-10, IL12p40, TNF-α and IL-6 cytokines were quantified using a multiplex bead-based platform (Life Technologies).

### Flow Cytometry

Briefly, non-specific FC binding sites on viable PBMC were blocked for 10 min at 4 °C, using anti-CD16/32 FcγR antibody (BD). After washing, the cells were incubated with a Life/Dead stain (Life Technologies) for 20 min to exclude dead cells from the analysis. T cells and cytokines were identified using anti-CD3, anti-CD4, anti-CD8, anti-IFN-γ, anti-IL-4, and anti-IL10. All samples were acquired using a MACSQuant (Miltenyi Biotec) and data were analyzed with the FlowJo V10 software package.

### RNA extraction, library preparation and sequencing

RNA preparation, library construction and sequencing were performed essentially as described previously^61^. Briefly, a pool of 30 salivary gland pairs from female *P. kandelakii* were dissected, placed in RNAlater and kept at 4 °C for 48 h before being transferred to −70 °C until RNA extraction. SG RNA was extracted and isolated using the Micro FastTrack mRNA isolation kit (Invitrogen, Grand Island, NY) per manufacturer’s instructions. The integrity of the total RNA was checked on a Bioanalyzer (Agilent Technologies, Santa Clara, CA). mRNA library construction and sequencing were done by the North Carolina State University, Genomic Sciences Laboratory. The SG library was constructed using the TruSeq RNA sample prep kit, v. 2 (Illumina Inc., San Diego, CA). The resulting cDNA was fragmented using a Covaris E210 (Covaris, Woburn, MA). Library amplification was performed using eight cycles to minimize the risk of over-amplification. Sequencing was performed on a HiSeq. 2000 (Illumina) with v. 3 flow cells and sequencing reagents. One lane of the HiSeq machine was used for this and eight other libraries, distinguished by bar coding. No cross-contamination of sequences were observed between libraries.

### Bioinformatic analysis

Customized bioinformatic analysis was performed as described elsewhere^61^. Reads were trimmed of low-quality regions (<10), and only those with an average quality of 20 or more were used, comprising a total of 19,431,950 high-quality reads. RPKM values were mapped to a spreadsheet (presented as Supplemental File 1) containing the assembled Pkan sequence names organized by categories as previously described^61^ and with columns highlighting their best match to GO, PFAM, KOG, REFSEQ-INVERTEBRATE, Smart, SWISSPROT databases and to a custom database obtained from a NCBI protein search using the key words “diptera [organism]”. The spreadsheet also contains information on the presence of predicted signal peptide and cleavage sites (Supplemental File 1). For multiple sequence alignment and phylogenetic analysis PkanSILK, PkanYLW, PkanSP15 had their predicted signal peptide signal (SignaIP-5.0 sever^62^) removed and resulting protein sequence entered into a Basic Local Alignment Search tool (BLAST) against nr and tsa_nr databases. Homologous sequences were included if E-value was below 1^−10^ and belonged to the sand fly genera, with the exception of PkanYLW where a homologue from *Drosophila* was used to root the tree. Multiple sequence alignment and identity/similarity matrix were constructed on MacVector (Version 15.5.3) with MUSCLE using PAM 200 profile. In order to determine the best method for estimating Maximum Likelihood phylogenetic trees, the “Find best protein Models” feature of MEGA7^57^ was utilized. Through this feature, it was determined that the best method for modeling phylogeny for PkanSILK was JTT + G + I, for PkanYLW was WAG + G and for PkanSP15 was WAG + G + I. For Gaps/Missing data treatment, a partial deletion option was utilized. Finally, the reliability of the trees was tested, by bootstrap method (N = 1000). Nodes with <70% reliability were removed.

### Data access

The raw reads were deposited on the Sequence Read Archive (SRA) of the National Center for Biotechnology Information (NCBI) under bioproject ID PRJNA576495, biosample accession SAMN12995167. A total of 5878 coding sequences and their translated protein products were deposited in the Transcriptome Shotgun Assembly portal of the NCBI to the DDBJ/EMBL/GenBank databases under the accession numbers (GIFK01000001 - GIFK01005878).

### Statistical analyses

Lines present at scatterplot represent the mean and standard error of the mean. Graphs and statistical significance were prepared and analyzed using GraphPad Prism Software 8.0. Unpaired t-test followed by Mann-Whitney test or a one-way ANOVA followed by Bonferroni multiple comparisons test were used to evaluate statistical significance among groups. A p value < 0.05 was considered statistically significant.

## Supplementary information

Supplementary information 1.

Supplementary information 2.
