# Supplementary material for: Immunity to vector saliva is compromised by short sand fly seasons in endemic regions with temperate climates
Source: Sci Rep. 2020 May 14;10:7990. doi: 10.1038/s41598-020-64820-9 (PMC7224377; doi:10.1038/s41598-020-64820-9)
Supplement: Supplementary file 1 — Supplementary information 1. [file 41598_2020_64820_MOESM1_ESM.pdf]

Oliveira_Pkan_Figure_S1

Immunity to vector saliva is compromised by short sand fly seasons in endemic regions with temperate climates

Fabiano Oliveira[1], Ekaterina Giorgobiani[&,2], Anderson B. Guimarães-Costa[1], Maha Abdeladhim[1], James Oristian[1], Lamzira Tskhvaradze[2], Nikoloz Tsertsvadze[2], Mariam Zakalashvili[2], Jesus G. Valenzuela[1*], and Shaden Kamhawi[1*]

[1]Vector Molecular Biology Section, Laboratory of Malaria and Vector Research, National Institute of Allergy and Infectious Diseases, National Institutes of Health, Rockville, Maryland 20852, USA.

[2] R. G. Lugar Center for Public Health Research, National Center for Disease Control and Public Health (NCDC), Kakheti Highway 99, 0198, Tbilisi, Georgia.

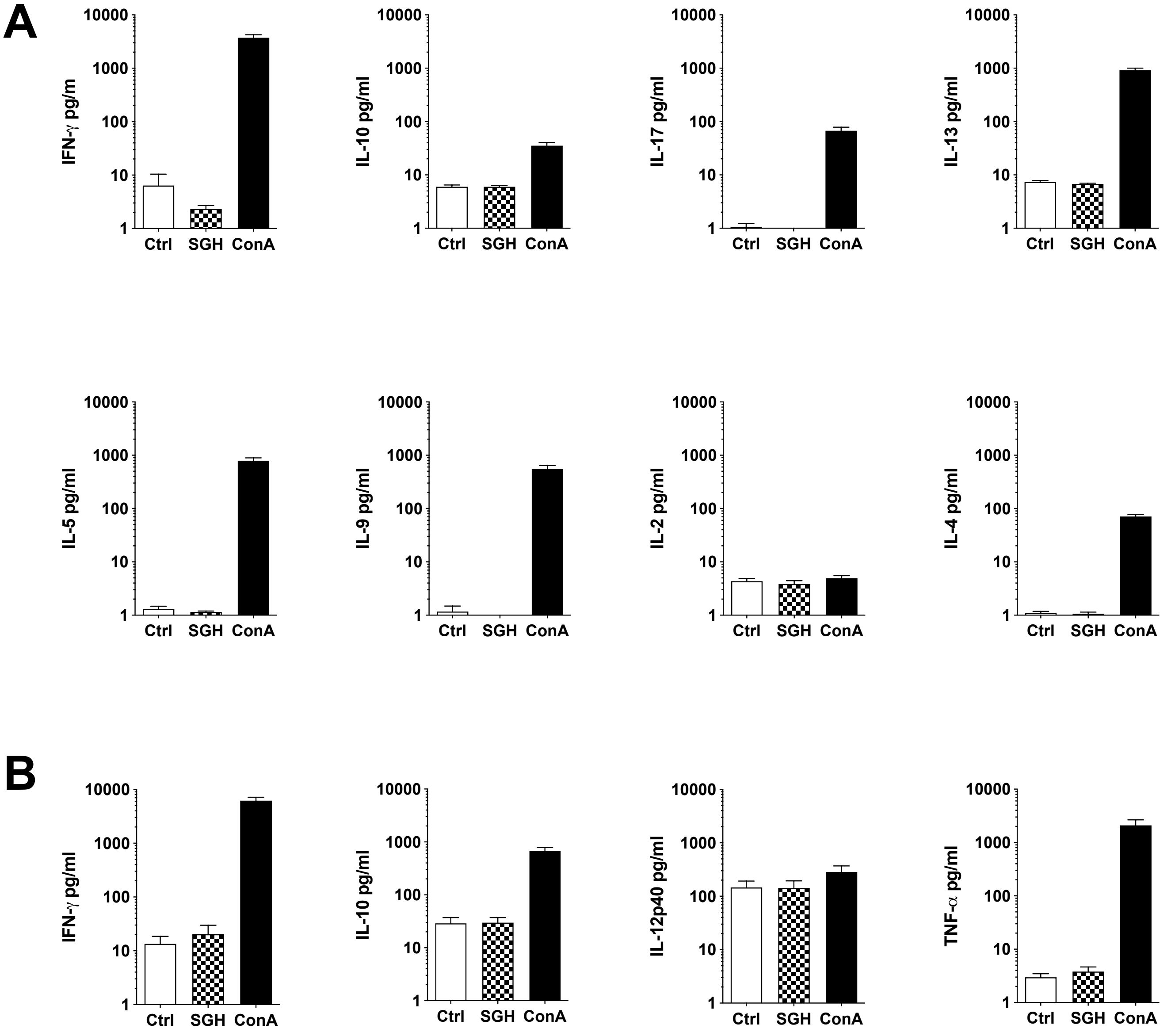

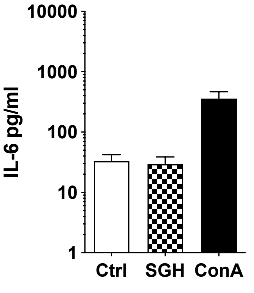

Supplemental Figure 1: (A) Human or (B) Dog PBMC were cultured unstimulated, stimulated with P. kandelakii SGH, or stimulated with Concavalin A (ConA) as a positive control for 96 hours. Supernatants were tested on a Luminex platform. Error bars represent the standard error of the mean.

Supplemental Table 1: Similarity and Identity matrix comparing PkanYLWs and sand fly related proteins.

	PkanYLWa	PkanYLWb	PkanYLWc	PkanYLWd	AHF49000.1P.perniciosus	ABA43050.1P.perniciosus	CCK33661.1P.perniciosus	AGT96428.1P.orientalis	ADJ54080.1P.tobbi	ADJ54079.1P.tobbi	AAX44093.1P.ariasi	AAX56360.1P.ariasi	AGT96460.1P.orientalis	AGT96427.1P.orientalis	ABA43049.1P.perniciosus	ABA12136.1P.argentipes	AGE83095.1P.papatasi	AGE83094.1P.papatasi	ARE29946.1P.papatasi	AAL11051.1P.papatasi	ARE29947.1P.papatasi	AAL11052.1P.papatasi	ABI15938.1P.duboscqi	ABI20172.1P.duboscqi	ABI15941.1P.duboscqi	ADJ54115.1P.sergenti	ADJ54116.1P.sergenti	ADJ54114.1P.sergenti	ADJ54122.1P.sergenti	ADJ54123.1P.sergenti	BAM69185.1L.ayacuchensis	BAM69110.1L.ayacuchensis	BAM69111.1L.ayacuchensis	BAM69109.1L.ayacuchensis	ANW11468.1B.olmeca	ANW11469.1B.olmeca	ANW11467.1B.olmeca	AAD32198.1L.longipalpis	AFP99235.1N.intermedia	JAV07968.1N.neivai	JAV07958.1N.neivai	JAV07960.1N.neivai	ABB00904.1L.longipalpis	AAS05318.1L.longipalpis	NP_523586.1D.melanogaster
PkanYLWa	100	95.2	98.7	81.3	80.2	80.9	80.1	88.5	86.4	74.3	85.3	81	77.8	77.3	76.2	61.7	53.4	53.3	43.6	53	43.9	52.9	52.3	52.1	51.8	55.1	54.6	55.1	54	53.4	56.3	54.3	54.3	53.8	52.5	56.6	51	46.7	56.3	56.2	53.6	53.2	49.2	52.3	22.2
PkanYLWb	97.6	100	96	86.1	82.8	83.5	82.4	90.4	88.3	73.3	87.2	79.2	76.5	76	75.5	63.5	55.7	53.5	43.9	53.3	44.1	55.2	54.2	53.4	53.1	55.6	55.6	55.4	54.6	55.2	57.9	53.5	53.5	53	51.4	58.1	50.8	47.5	57.6	57.5	54.6	52.7	49.5	52.1	22
PkanYLWc	99.5	97.6	100	82.1	80.2	80.9	80.1	88.5	86.4	74.1	85.6	80.7	77.5	77	75.9	61.9	53.6	53.3	43.6	53	43.9	53.1	52.6	52.3	52.1	55.1	54.3	54.8	54	53.6	56.3	53.8	53.8	53.2	51.7	56.8	50.5	46.4	56.3	56.2	53.6	53	48.7	51.6	22
PkanYLWd	88.3	90.7	88.3	100	71.9	72.8	71.5	78.6	76.8	82.2	78.1	82.7	86.3	85.7	84.4	61.1	52.4	52.2	43.8	52.2	43.8	51.6	51.7	52.1	52.1	55.7	57	55.4	56.2	52.6	54.8	51.6	51.6	50.8	49.6	54.3	49.7	44.9	54	54.1	51.3	51.7	50.3	51.6	21.1
AHF49000.1P.perniciosus	89.9	91.3	89.7	84.1	100	98.4	98.7	84.9	89.7	68.7	78.8	68.7	69.5	69	69.5	60.2	52.7	48.8	39.7	48.6	40.5	52.7	52.1	50.3	50	52.5	52.7	52.2	51.4	54	55.2	51.7	51.7	51.2	49.6	54.6	48.7	46.8	53.3	53.2	50.4	49.9	48.2	49.2	22.8
ABA43050.1P.perniciosus	90.4	91.8	90.2	84.5	99.5	100	98.1	86.1	90.1	69.3	80.1	69.3	70.1	69.6	70.1	61	53.2	49.3	40.2	49.1	41	53.2	52.3	50.5	50.3	53	53	52.7	52	54.5	55.2	52.2	52.2	51.7	50.1	54.4	49.2	47.1	53.6	53.5	50.6	50.4	48.7	49.7	22.6
CCK33661.1P.perniciosus	90.7	91.5	90.4	84.3	99.2	99.7	100	84.5	89.3	68.5	78.7	68.5	69.3	68.8	69.3	59.9	52.7	48.6	39.4	48.3	40.2	52.7	52.1	50.3	50	52.5	52.7	52.2	51.4	54	54.9	51.7	51.7	51.2	49.6	54.4	48.7	46.6	53.1	52.7	50.1	49.9	48.2	49.2	22.8
AGT96428.1P.orientalis	95.5	95.7	94.9	87.4	92.6	93.1	92.8	100	90.9	75.1	84	77.7	76.9	76.4	76.4	61.4	54.4	53.1	43.2	52.9	44	54.4	53.3	53.5	53	56.3	55.8	56	55.2	55.5	57.4	53.4	53.4	52.9	51.6	57.9	50.9	47.2	57.3	57.2	54.3	53.1	49.3	52.5	23.3
ADJ54080.1P.tobbi	93.1	94.4	92.6	86.9	94.4	94.9	94.7	94.9	100	71.5	81.4	73.3	73.6	73.1	72.5	59.4	53.5	52	42	51.7	42.3	53.5	53.4	51.6	51.3	54	53.5	53.8	53	53.5	55.4	51.7	51.7	51.2	49.9	55.2	49.5	45.3	53.3	53.2	50.4	50.9	47.9	50.3	22.8
ADJ54079.1P.tobbi	84	82.9	83.7	89.8	81.2	81.6	81.3	83.9	82.4	100	71.5	80.5	87	86.8	87.3	56.8	49.5	48.8	41.4	48.8	41.7	49	49.6	49.2	49.2	53	53.8	53	53.3	50	49.9	50.3	50.3	49.5	48	49.4	47.6	41.9	49.6	49.5	47.2	49.3	50.5	49.7	22.6
AAX44093.1P.ariasi	92	93.1	91.7	86.9	89.2	89.6	89.9	91.2	89.4	81.6	100	76.5	74.7	74.1	76	62.5	54.2	52.7	43.1	52.5	43.9	53.6	53.1	52.3	52.1	55.6	55.9	55.4	54.6	54.9	55.8	51.7	51.7	51.2	50.1	54.8	50.8	46.7	55.3	55.2	52	50.9	49.2	50.8	22.9
AAX56360.1P.ariasi	86.9	85.9	86.6	89.8	80.6	81.1	81.3	86.1	83.5	88.4	84.3	100	83.8	83.5	82.4	56.6	51	51.7	43.3	51.7	43.3	50.5	50.7	51.1	51.1	55.1	55.4	54.9	55.7	51	51.7	50.5	50.5	49.7	48.6	51.4	49.2	43.1	51.2	51.3	48.7	50.4	48.4	50.8	21.7
AGT96460.1P.orientalis	86.6	85.1	86.4	92.5	82.2	82.7	82.9	86.1	84.5	91.9	84	90.5	100	99.5	88.1	57.9	50	50.5	42.3	50.5	42.1	49.2	49.9	50.4	50.7	55	55	54.5	55.3	51	51.7	51	51	50.3	49.3	50.6	48.3	42.9	50.4	50.5	48	51.2	49.7	51	22.2
AGT96427.1P.orientalis	86.1	84.5	85.8	91.9	81.7	82.1	82.4	85.5	84	91.9	83.5	90.3	99.5	100	87.6	57.6	49.7	50.3	42.1	50.3	41.8	49	49.6	49.9	50.1	54.5	54.5	54	54.8	50.8	51.4	50.8	50.8	50	48.8	50.4	48.5	42.4	50.1	50.3	47.7	50.7	49.5	50.5	22.2
ABA43049.1P.perniciosus	84.5	84	84.2	90.8	81.2	81.6	81.9	84.7	83.2	90.8	83.7	88.1	91.9	91.4	100	57.4	50.5	50.7	42.7	50.7	43.3	49.7	50.7	51.6	51.6	55.1	55.7	54.9	55.4	52.3	53.5	50.3	50.3	49.5	48.3	51.2	48.4	43.9	51.4	51.5	48.7	49.3	50.5	49.7	22
ABA12136.1P.argentipes	78.5	79.5	78.2	77.9	77.1	77.5	77.5	78.2	77.5	74.5	78.5	73.9	75.3	75	74.5	100	55.4	49	41.7	49.5	42.2	54.9	53.9	49.4	49.6	53.4	52.3	52.6	51.6	53.5	54.1	52.5	52.5	51.9	48.3	52.1	46.4	46.6	52	52.4	50.1	50.1	46.6	51.5	21.8
AGE83095.1P.papatasi	67.2	69.3	66.9	68.3	66.9	67.3	67	68.5	69.4	65.7	68.5	66.8	65.2	65.2	66.8	68.7	100	56	47.6	56.5	47.9	99	88.2	55.4	55.9	61	59.4	61.3	59.7	76.8	52.4	54.3	54.5	53.5	50.6	50.1	49.5	46.7	50.6	50	48.2	49.4	45.6	49.7	22.3
AGE83094.1P.papatasi	67.6	67.6	67.1	67.3	65.5	65.8	66.1	68.1	68.7	65.7	68.1	67.3	66.7	66.7	67.3	65.5	71.7	100	79.8	99.2	77.7	55.5	56.2	83.3	83	74.8	68.7	74.3	71.9	58.1	47.4	48.6	48.6	48.1	46.6	48.7	46.7	45.8	49.5	49.1	47.4	46.9	43.1	49.1	23
ARE29946.1P.papatasi	55.1	55.9	54.6	55.7	53.8	54	54.3	56	56.7	53.8	55.9	55.4	54.5	54.5	55.1	54.9	60.2	80.9	100	80.1	92.7	47.1	46.7	68.7	68.2	60.5	56.2	59.9	58.6	49	39.7	41	41	40.5	37.8	40.8	38.2	39.3	41.3	41.2	41.1	38.8	36.8	40.6	19
AAL11051.1P.papatasi	67.4	67.4	66.8	67	65.2	65.5	65.8	67.8	68.4	65.4	67.9	67	66.4	66.4	67	65.8	72	99.7	80.9	100	77.9	56	56.4	83.8	83.6	75.3	69.2	74.8	72.4	58.9	47.9	48.8	48.8	48.3	46.9	48.5	47	46.3	49.5	49.6	47.9	47.1	42.8	49.4	22.6
ARE29947.1P.papatasi	55.9	56.7	55.4	56.5	54.3	54.6	54.8	56.5	56.9	54.1	56.4	55.7	54.8	54.8	55.1	55.7	60.5	79.5	93.9	79.5	100	47.4	47	68.4	67.9	61	56.8	61	59.7	49.5	40.5	42.1	42.1	41.6	38.5	41.3	38.9	40.1	41.8	41.7	41.4	39.8	37.6	41.4	18.4
AAL11052.1P.papatasi	67.2	69.3	66.9	68.1	66.9	67.3	67	68.5	69.4	65.7	68.5	66.8	64.9	64.9	66.5	68.7	99.5	72	60.5	72.3	60.7	100	87.4	54.8	55.4	60.5	58.9	60.7	59.2	76.3	52.2	53.7	54	53	50.6	50.1	49	46.7	50.6	50	48.2	48.8	45.1	49.2	22.1
ABI15938.1P.duboscqi	67.4	69.3	67.2	69.6	67.4	67.7	67.2	68.9	69.3	66.4	68.8	68.2	67.5	67.5	68.2	68.1	92.1	71.4	59.3	71.7	59.6	91.9	100	56	56.8	61.7	59.1	61.9	60.1	75.8	50.9	54.7	54.9	53.9	51.3	48.6	49.6	46.5	48.1	47.7	45.7	49	44.7	49.4	23
ABI20172.1P.duboscqi	66.4	67.2	66.1	66.6	66.1	66.4	66.4	67.4	68	63.9	67.7	65.5	65.4	64.9	66.6	64.6	71	89.7	73.7	89.7	72.9	71.3	70.4	100	98.4	74.9	70.1	74.6	72.5	58.2	49.1	50.3	50.3	49.5	48.6	48.8	47.4	46.5	49.1	49	47.7	48.6	44.5	49	22.1
ABI15941.1P.duboscqi	66.1	66.9	65.9	66.6	66.1	66.4	66.4	67.1	68	63.9	67.4	65.5	65.7	65.2	66.6	65.1	71.5	89.7	73.5	89.7	72.7	71.8	70.9	98.4	100	74.6	69.8	74.3	72.2	58.4	49.4	50.3	50.3	49.5	48.6	48.6	46.9	46.7	48.6	48.5	47.2	48.3	44	49.2	22.3
ADJ54115.1P.sergenti	69.7	70.2	69.5	71	68.3	68.7	68.7	69.6	70.5	67.3	71.5	69.9	69.8	69.3	70.2	67.9	76.4	86.5	69.8	86.5	69.8	76.7	75.9	85.7	86	100	87.3	97.6	95	65.1	53.8	55.1	55.1	54.3	52.9	51.3	49.2	49.1	51.5	51.9	50.4	53.1	46	52.5	23.5
ADJ54116.1P.sergenti	69.2	70.8	68.9	72	69.6	70	69.7	70.2	70.5	67.5	72.1	69.7	69.3	68.8	70.2	68.7	75.9	79.6	65	79.6	65	75.9	74.5	79.9	79.9	92	100	87	89.7	61.5	52.3	54.5	54.5	53.8	52.9	50	48.4	46.9	50.3	50.6	48.9	52.9	48	52.2	22.8
ADJ54114.1P.sergenti	69.7	70.2	69.5	71	68.3	68.7	68.7	69.6	70.5	67.3	71.5	69.9	69.6	69	70.2	67.9	77	86.2	69.5	86.2	69.5	77.2	76.4	85.4	85.7	99.7	92.3	100	94.2	65.4	52.8	54.8	54.8	54	53.1	50.8	48.2	49.1	50.5	50.9	49.4	52.9	46.2	52.2	23
ADJ54122.1P.sergenti	70	70.5	69.7	71.8	68.6	68.9	68.9	69.9	70.8	68.1	72.1	70.4	70.6	70.1	71	67.6	76.7	84.6	69	84.6	69	77	76.1	83.6	83.9	97.9	93.6	97.6	100	63.3	52.6	54.3	54.3	53.5	51.8	49.7	49.5	48.4	50	50.4	48.9	52.6	47	51.4	23
ADJ54123.1P.sergenti	70.1	71.1	69.8	69.8	69.8	70.1	69.6	70.8	70.6	68	71.6	68.5	68.2	68.2	69.8	69.3	85.7	74	60.9	74.2	60.9	85.7	84.4	71.9	72.5	78.1	76	78.1	77.6	100	52.7	51.9	51.9	50.9	49.6	50.9	49.2	47.7	49.6	49.5	48	48.3	45.7	48.5	21.7
BAM69185.1L.ayacuchensis	68.5	69.8	68	68	68.3	68.3	68.3	69.3	68.6	64.6	67.2	66.1	65.4	65.1	66.4	68.5	64.2	62.1	52.1	62.1	53.1	63.9	63.7	62.7	62.7	65.4	65.9	65.6	65.1	64.7	100	54.5	54.5	53.5	50.6	72.6	48.8	67	74.3	74.5	70.6	52.4	46.1	52.3	21.7
BAM69110.1L.ayacuchensis	69.1	69.9	68.6	68.6	69.5	69.6	69.9	69.5	69.6	67.8	70.1	66.8	67.5	67.5	66.2	69.3	69.3	66	54.8	66	55.6	69.3	69.7	66.3	66.3	68.6	68.6	68.6	68.6	67.6	70.7	100	99.7	98.4	78.8	54	62	49.7	52.7	53.3	51	77.7	62.3	78.9	23.2
BAM69111.1L.ayacuchensis	69.1	69.9	68.6	68.6	69.5	69.6	69.9	69.5	69.6	67.8	70.1	66.8	67.5	67.5	66.2	69.3	69.5	66	54.8	66	55.6	69.5	69.9	66.3	66.3	68.6	68.6	68.6	68.6	67.6	70.7	99.7	100	98.7	79.1	54	62	49.7	52.7	53.3	51	78	62.6	79.1	23.2
BAM69109.1L.ayacuchensis	68.6	69.1	68.1	67.5	68.7	68.8	69.4	68.8	68.8	66.8	69.6	66	66.8	66.8	65.4	68.7	68.5	65.5	54.3	65.5	55.1	68.5	68.9	65.5	65.5	67.8	67.8	67.8	67.8	66.6	69.7	98.4	98.7	100	78.3	53	60.7	49	51.7	52.3	50	76.7	61.5	77.8	23.2
ANW11468.1B.olmeca	67	67.8	66.5	67.2	67.2	67.3	67.5	67.4	67.3	65.1	68.6	65.1	65.1	64.6	64	66.7	67.2	64.8	53.6	64.8	53.9	67.2	67.1	64.7	64.9	67.7	67.4	67.4	67.4	66.1	66.6	88	88.2	87.4	100	51.7	59.8	46.5	47.8	47.9	46.7	79.7	57.2	70.4	22.1
ANW11469.1B.olmeca	72.1	73.1	71.6	70.3	70.1	70.1	70.1	72.1	70.6	66.4	70.5	67.2	66.1	65.9	66.4	69.5	67	62.1	51.8	62.1	52.6	67.3	66	62.1	61.9	64.1	64.9	64.1	64.1	67.3	84.2	71	71	69.9	68.9	100	48.1	60.7	78.9	78.4	74.4	52.7	48.2	50.5	20.8
ANW11467.1B.olmeca	64.1	65.1	63.8	65.3	64	64.3	64.3	65	63.5	63.9	65.9	64.7	64.1	64.6	63.7	64.5	64.2	65.4	54.5	65.6	54.5	64.2	64.2	65.4	64.7	64.9	64.9	64.7	65.4	64.2	65.3	77.2	77.2	75.9	75.6	66.1	100	43.4	50.6	51.3	48.7	59.1	53.5	57.6	21
AAD32198.1L.longipalpis	65.7	66.5	65.2	64.5	65.3	65.3	65.1	65.7	65.3	62.4	64.7	61.9	62.2	61.7	62.2	65.1	61.8	60.7	50.9	60.7	52.4	62.1	61.8	60.8	60.8	63.5	62.7	63.5	63	63.6	78.7	65.2	65.2	64.1	60.9	76.4	58.8	100	62	62.4	58.2	46.5	42.3	47.4	20.5
AFP99235.1N.intermedia	70.4	71.5	69.9	69.2	68.5	68.5	68.2	71	69	65.8	68.9	66.6	66.1	65.8	65.3	67.3	65.9	64.8	53.8	64.8	54.6	66.2	64.9	64.6	64.1	65.1	65.1	64.8	65.1	66.2	83.8	69.3	69.6	68.5	66	87.1	67.5	77.7	100	97.7	93.4	49.6	46.2	49.7	22.1
JAV07968.1N.neivai	70.6	71.6	70.1	69.3	68.6	68.6	68.4	71.1	69.2	65.7	68.8	66.8	66.2	66	65.5	67.5	65.1	64.7	53.7	64.7	54.5	65.3	64.3	64.3	63.8	65.2	65	65	65.2	65.8	84.3	70	70.3	69.2	66.2	86.9	68.5	77.9	98.5	100	92.4	50.3	46	50.4	21.2
JAV07958.1N.neivai	67.5	68.5	67	66.2	65.3	65.3	65.1	67.8	65.8	62.7	65.7	63.2	62.9	62.7	62.2	65	63.1	63.2	53.8	63.2	54.1	63.3	62.3	63.3	62.8	63.7	63.5	63.5	63.7	63.6	79.9	67.9	68.2	67.2	64.4	82.7	65.9	74	94.4	93.7	100	48	44.6	48.5	21.9
JAV07960.1N.neivai	66.8	67.3	66.2	66.9	65.9	66	66.2	67.4	66.8	65.1	67.3	65.6	65.6	65.1	64.3	67.4	66.4	64.1	53.4	64.1	54.2	66.7	65.8	63.9	64.2	67.4	67.2	66.9	66.9	64.5	68.6	89	89.3	88	89.2	67.9	74	61.9	66.5	67.2	64.4	100	57.7	73.3	21.5
ABB00904.1L.longipalpis	62.5	63.8	62.2	65	61.9	62.2	62.2	62.7	62	65.5	63.8	61.6	63.2	63.2	64.7	63.7	61.1	60.3	50.1	60.3	50.4	60.9	60.8	60.9	60.2	62.4	63.2	62.7	63.2	61.5	63.4	75.7	75.9	74.9	71.7	62.1	70.3	58.5	62.3	63.2	60.8	72.7	100	59.4	22.4
AAS05318.1L.longipalpis	67.9	68.4	67.4	68.1	68.6	68.7	68.9	68.8	68.7	67.5	68.4	67	67.5	67	66	69.3	65.2	64.9	53.9	64.9	54.2	65.2	64.6	65.8	66.1	67.8	67.8	67.5	67.5	64.6	69.7	89.6	89.8	88.5	83.5	67.9	72.8	64.7	68.1	68.8	66.4	84.6	73.6	100	22.3
NP_523586.1D.melanogaster	38.1	38.6	38.1	38.1	39.1	38.9	38.9	39	38.7	38.1	38.6	38.6	38.1	38.3	37.4	36.5	36.5	37.4	31.4	37.4	30.8	36.7	36.3	36.4	36.4	36.3	37.2	36.1	35.8	37.6	35.9	37.8	37.8	37.8	36.1	35.5	37.6	35.4	38.3	38.1	37.2	36.5	34.1	37.3	100

% of identity

% of similarity
